# P-754. Assessment of a Staphylococcus aureus Polymerase Chain Reaction (PCR) Wound Swab for Early Antimicrobial Modification Across Four Community Hospitals

**DOI:** 10.1093/ofid/ofaf695.965

**Published:** 2026-01-11

**Authors:** Michael Dickens, Sheila K Wang, Rishita Shah, Jaime Borkowski, Kyle Johnicker, Radhika S Polisetty

**Affiliations:** Northwestern Medicine, Carpentersville , IL; Midwestern University College of Pharmacy/Northwestern Memorial Hosptial, Downers Grove, Illinois; Northwestern Lake Forest Hospital, Lake Forest, IL, Illinois; NM Delnor Hospital, Geneva, Illinois; Northwestern Medicine Kishwaukee Hospital, DeKalb, Illinois; Midwestern University College of Pharmacy/ Northwestern Medicine Central DuPage Hospital, Winfield, Illinois

## Abstract

**Background:**

While the utilization of nasal PCR for de-escalation of methicillin resistant Staphylococcus aureus (MRSA) therapy is well described, there is less evidence to describe the performance and utility of a directed Staphylococcus aureus (SA) PCR for skin infections (SSTIs). Our study provides evidence of the performance of a MRSA/SA PCR wound swab and its implication for antimicrobial stewardship.

**Figure:**
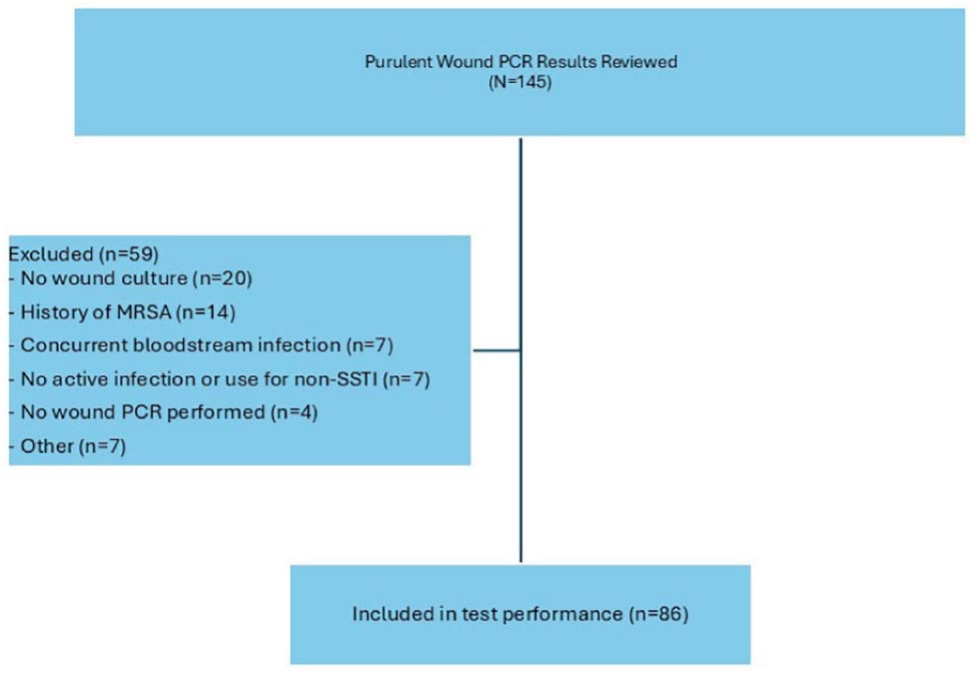
Inclusion and Exclusion

**Figure:**
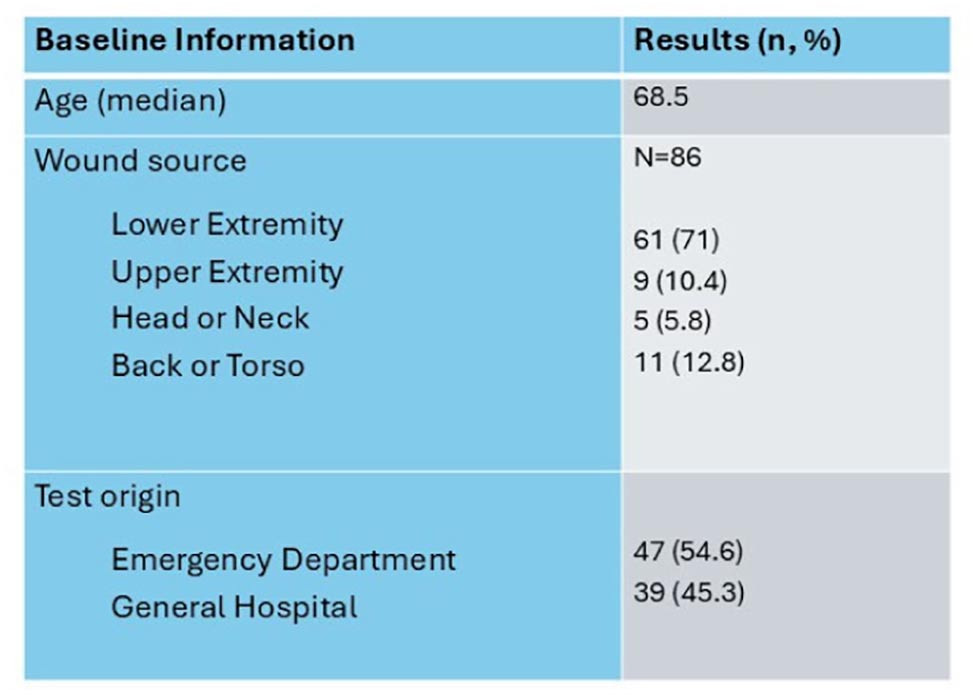

**Methods:**

Lab orders for the MRSA/SA PCR wound swab were generated using an internal query tool embedded into the electronic health record of the Northwestern Medicine health system (for four hospitals). Patients eligible for inclusion: 1) adult patients ≥18 years of age, 2) received inpatient treatment for a wound infection at one of four internal institutions serviced by our regional microbiology lab, 3) had a wound culture and wound PCR obtained between 11/1/2025 and 4/1/2025. Key exclusion criteria were patients who 1) did not receive treatment for infection, 2) had known history of MRSA within the previous two years, 3) had any positive blood culture during admission. The primary objective of the study was to determine the median time to appropriate antimicrobial therapy based on PCR test results. Secondary objectives were to determine the accuracy of the MRSA/SA PCR wound swabs across the four community hospitals in the setting of both monomicrobial and polymicrobial cultures.

**Figure:**
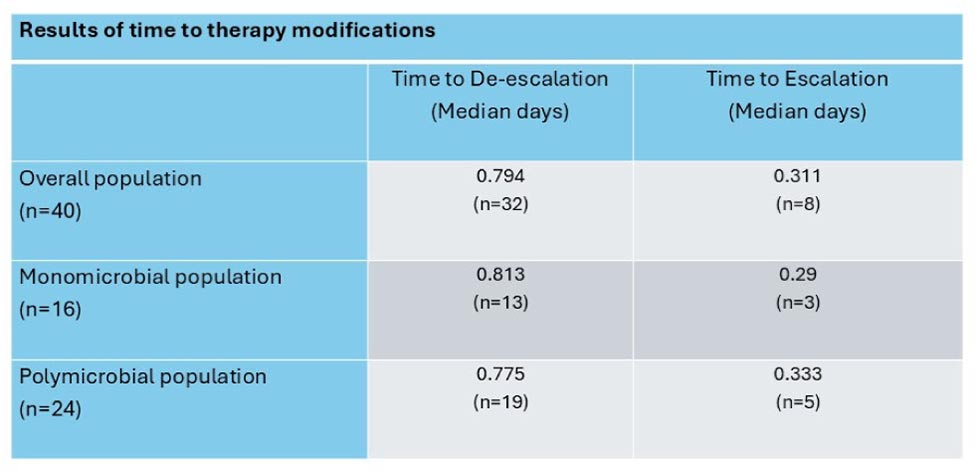

**Figure:**
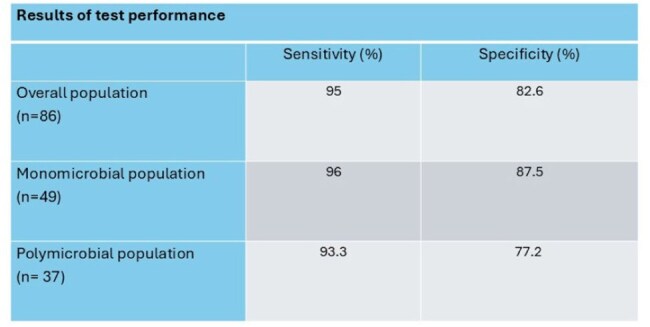

**Results:**

After screening, 86 patients were included. Of those included, 40 (47%) had antimicrobial therapy appropriately de-escalated (n=32) or escalated (n=8) based on the PCR result. The median time to de-escalation was 0.79 days, whereas the median time to escalation was 0.31 days. The test performance resulted in a good overall accuracy with a sensitivity of 95% and a specificity of 83%, with numerically higher performance observed in monomicrobial wound cultures compared to polymicrobial cultures.

**Conclusion:**

The use of a MRSA/SA wound PCR provided highly sensitive and specific information that led to appropriate therapy modifications in nearly half of patients included in this study. A MRSA purulent wound PCR may serve as a valuable tool for antimicrobial stewardship to leverage de-escalation efforts, though careful patient selection may be required, especially in the setting of polymicrobial wounds.

**Disclosures:**

All Authors: No reported disclosures

